# Randomized-controlled trial assessing a digital care program versus conventional physiotherapy for chronic low back pain

**DOI:** 10.1038/s41746-023-00870-3

**Published:** 2023-07-07

**Authors:** Di Cui, Dora Janela, Fabíola Costa, Maria Molinos, Anabela C. Areias, Robert G. Moulder, Justin K. Scheer, Virgílio Bento, Steven P. Cohen, Vijay Yanamadala, Fernando Dias Correia

**Affiliations:** 1 Physical and Rehabilitation Medicine, Emory University, Atlanta, GA Georgia; 2 Sword Health, Inc., Draper, UT USA; 3 Institute for Cognitive Science, University of Colorado Boulder, Boulder, CO USA; 4 Department of Neurological Surgery, University of California, San Francisco, CA USA; 5 Departments of Anesthesiology & Critical Care Medicine, Physical Medicine and Rehabilitation, Neurology, and Psychiatry and Behavioral Sciences, Johns Hopkins School of Medicine, Baltimore, MD USA; 6 Departments of Anesthesiology and Physical Medicine and Rehabilitation and Anesthesiology, Uniformed Services University of the Health Sciences, Bethesda, MD USA; 7 Department of Surgery, Quinnipiac University Frank H. Netter School of Medicine, Hamden, CT USA; 8 Department of Neurosurgery, Hartford Healthcare Medical Group, Westport, CT USA; 9 Neurology Department, Centro Hospitalar e Universitário do Porto, Porto, Portugal

**Keywords:** Randomized controlled trials, Orthopaedics, Chronic pain, Rehabilitation

## Abstract

Low back pain (LBP) is the world’s leading cause of years lived with disability. Digital exercise-based interventions have shown great potential in the management of musculoskeletal conditions, promoting access and easing the economic burden. However, evidence of their effectiveness for chronic LBP (CLBP) management compared to in-person physiotherapy has yet to be unequivocally established. This randomized controlled trial (RCT) aims to compare the clinical outcomes of patients with CLBP following a digital intervention versus evidence-based in-person physiotherapy. Our results demonstrate that patient satisfaction and adherence were high and similar between groups, although a significantly lower dropout rate is observed in the digital group (11/70, 15.7% versus 24/70, 34.3% in the conventional group; *P* = 0.019). Both groups experience significant improvements in disability (primary outcome), with no differences between groups in change from baseline (median difference: −0.55, 95% CI: −2.42 to 5.81, *P* = 0.412) or program-end scores (−1.05, 95% CI: −4.14 to 6.37; *P* = 0.671). Likewise, no significant differences between groups are found for secondary outcomes (namely pain, anxiety, depression, and overall productivity impairment). This RCT demonstrates that a remote digital intervention for CLBP can promote the same levels of recovery as evidence-based in-person physiotherapy, being a potential avenue to ease the burden of CLBP.

## Introduction

Low back pain (LBP) is a major health problem^1^, considered the leading cause of years lived with disability^1^ and of absenteeism. Although the impact on productivity varies in the literature^2^, one systematic review estimated the direct medical costs as $300 billion in the United States (U.S.) alone^3^. Unsatisfactory LBP management may lead to overutilization of imaging^4^, surgeries^5,6^, and medication, including opioids^7,8^.

Current guidelines for chronic LBP (CLBP) management recommend physiotherapy as a first-line intervention, alongside education and behavioral interventions^9,10^. Moderate-certainty evidence from randomized controlled trials (RCTs) supports the effectiveness of exercise-based physiotherapy in reducing pain and disability in LBP treatment^11^, and these interventions have often yielded better outcomes for disability and return to work than surgical interventions^2,12^.

However, access to in-person physiotherapy faces several barriers: a scarcity of healthcare resources (including therapists and facilities), time-, travel-, and costs-constraints (work time off, childcare costs), insufficient health literacy, and, more recently, the perceived risk of contracting infections^13^. All these also affect engagement, resulting in high percentages of unattended or incomplete treatments^14^.

Digital interventions have great potential in overcoming such challenges, being more accessible and affordable than in-person physiotherapy^15,16^, and increasing patient adherence and empowerment^17^. Within LBP management, research has focused on the effectiveness and safety of digital interventions, both as adjuncts to in-person care^18,19^ and as stand-alone through video conference-based^19^ or asynchronous telerehabilitation^20,21^. The latter has the potential to scale care delivery, addressing the growing prevalence of CLBP^1^. However, the few trials comparing exercise-based asynchronous interventions with standard in-person physiotherapy considered cohorts with diverse acuity levels^20,21^ or were non-randomized studies^20^, compromising the certainty of evidence on the subject. Thus, further research is needed on the effectiveness of these solutions as an alternative to in-person physiotherapy for CLBP.

Previously, we demonstrated the effectiveness of tailored digital care programs (DCP) integrating exercise, education, and cognitive behavioral therapy (CBT) in several musculoskeletal conditions^22,23^, including acute and chronic LBP^24,25^. The present RCT aims to compare the clinical outcomes of patients with CLBP following a DCP versus conventional in-person physiotherapy. We hypothesize that outcomes are comparable to those obtained with conventional physiotherapy.

## Results

**Fig. 1:**
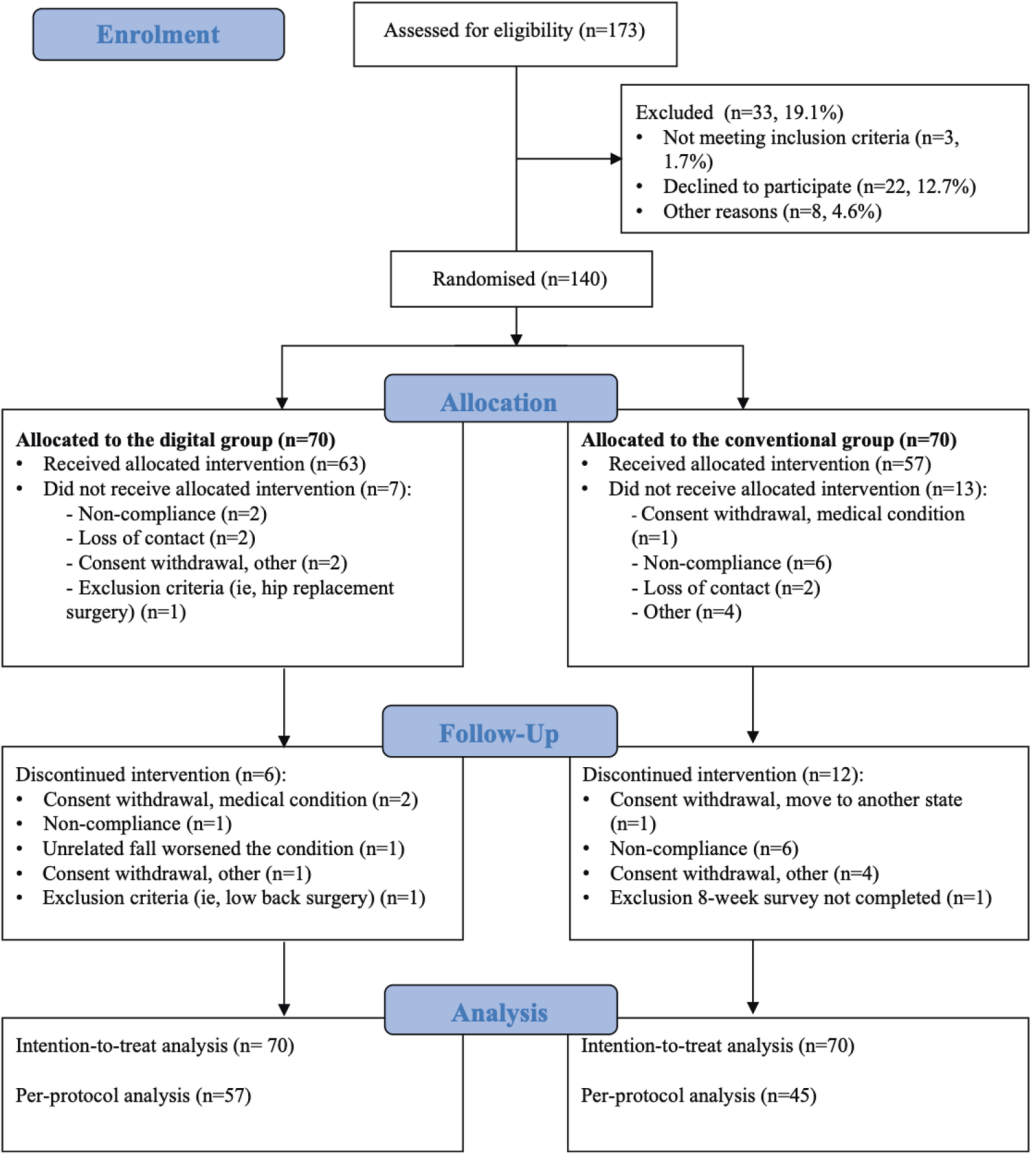
Study flow diagram.

Eligibility screening was conducted for 173 participants: 22 declined participation, 3 were not eligible, and 8 were excluded. In total, 140 participants were randomly assigned to the digital group (DG) or conventional group (CG) (*N* = 70, each). The completion rate was 81.4% (57/70) in the DG and 64.3% (45/70) in the CG (Fig. 1).

### Baseline characteristics

Baseline demographics were similar between groups within intention-to-treat (ITT) analysis (*N* = 140, Table 1). In per-protocol analysis (*N* = 102), no differences were observed except for age, with younger participants in the DG (Supplementary Table 1). Comparing completers (*N* = 102) with non-completers (*N* = 38), participants with lower levels of education, smokers, and those who reported not exercising at baseline were less likely to complete the program (Supplementary Table 2).

**Table 1:** Baseline characteristics of study participants (*N* = 140).

Characteristic	Digital Group (*N* = 70)	Conventional Group (*N* = 70)	P*
Age (years), median (IQR)	50.50 (22.00)	54.50 (20.00)	0.411
*Age categories (years), N (%)*			0.077
<25	1 (1.4)	4 (5.7)	
25–40	21 (30.0)	10 (14.3)	
41–60	30 (42.9)	31 (44.3)	
>60	18 (25.7)	25 (35.7)	
*Gender, N (%)*			0.587
Woman	46 (65.7)	49 (70.0)	
Man	24 (34.3)	21 (30.0)	
BMI, median (IQR)	28.26 (9.29)	28.30 (8.22)	0.709
*BMI categories, N (%)*			0.977
Normal (18.5–25)	21 (30.0)	21 (30.0)	
Overweight (>25–30)	21 (30.0)	22 (31.4)	
Obese (>30–40)	23 (32.9)	21 (30.0)	
Morbidly obese (>40)	5 (7.1)	6 (8.6)	
*Race, N (%)*			0.391
Asian or Pacific Islander	11 (15.7)	6 (8.6)	
Black or African American	27 (38.6)	34 (48.6)	
Hispanic or Latino	3 (4.3)	2 (2.9)	
Native American or Alaskan Native	0 (0.0)	1 (1.4)	
White or Caucasian	28 (40.0)	23 (32.9)	
Multi-racial or biracial	0 (0.0)	2 (0.0)	
Prefer not to answer	1 (1.4)	2 (2.9)	
*Education level, N (%)*			0.870
Did not attend school	0 (0.0)	1 (1.4)	
Some high school	2 (2.9)	2 (2.9)	
High school graduate or GED	7 (10.0)	5 (7.1)	
Some colleges (some community colleges, associate degree)	17 (24.3)	19 (27.1)	
Four-year college degree or bachelor’s degree	17 (24.3)	14 (20.0)	
Some postgraduate or professional schooling, no postgraduate degree	4 (5.7)	6 (8.6)	
Postgraduate or professional degree (including master’s, doctorate, medical or law degree)	23 (32.9)	22 (31.4)	
Prefer not to answer	0 (0.0)	1 (1.4)	
*Employment status, N (%)*			0.638
Employed (part-time or full-time)	48 (68.6)	46 (65.7)	
Unemployed (seeking opportunities)	7 (10.0)	4 (5.7)	
Not employed and not seeking work	14 (20.0)	18 (25.7)	
Prefer not to answer	1 (1.4)	2 (2.9)	
*Exercise levels, N (%)*			0.358
None	10 (14.3)	14 (20.0)	
Less than 1 h	20 (28.6)	13 (18.6)	
Between 1–2.5 h	18 (25.7)	24 (34.3)	
>2.5 h	22 (31.4)	19 (27.1)	
*Comorbidities, N (%)*			
High blood pressure	23 (32.9)	24 (34.3)	0.858
High blood sugar or diabetes	8 (11.4)	8 (11.4)	1.00
Cardiac conditions	5 (7.1)	2 (2.9)	0.245
Respiratory conditions	10 (14.3)	10 (14.3)	1.00
None of the listed	38 (54.3)	37 (52.9)	0.865
Smoking habits, *N* (%)	1 (1.4)	3 (4.3)	0.310
Low back-related leg pain, *N* (%)	34 (48.6)	41 (58.6)	0.236
*Laterality of leg pain, N (%)*			0.601
Right	13 (38.2)	16 (39.0)	
Left	14 (41.2)	13 (31.7)	
Both	7 (20.6)	12 (29.3)	
Lumbar radicular pain, *N* (%)	21 (30.0)	23 (32.9)	0.716
Previous physiotherapy, *N* (%)	38 (54.3)	37 (52.9)	0.865
Previous or scheduled low back surgery, *N* (%)	9 (12.9)	6 (8.6)	0.412

Abbreviations: *BMI* body mass index, *GED* general educational development (includes technical or vocational training).

^*^Mann–Whitney *U* test or Pearson Chi-Square.

### Patient engagement

Treatment dosage was similar between groups, reflected by the similar time dedicated to exercise sessions (DG: 451.78, IQR 227.36 and CG: 385.98, IQR 145.36; *P* = 0.662, Table 2). On average, participants performed 22.32 (SD 9.46) exercise sessions in the DG and 12.42 (SD 4.95) in the CG. The dropout rate was higher in the CG (24/70, 34.3% vs. 11/70, 15.7%; *P* = 0.019).

**Table 2:** Engagement metrics of participants.

Engagement variable mean (SD)		*N*	Digital group	*N*	Conventional group	P*
Total sessions	ITT	63	22.32 (9.46)	57	12.42 (4.95)	
	PP	57	23.56 (9.01)	45	14.58 (2.24)	
Frequency of sessions per week	ITT	63	2.79 (1.18)	57	1.55 (0.62)	
	PP	57	2.95 (1.13)	45	1.82 (0.28)	
Total time during sessions	ITT	63	451.78 (227.36)	57	385.98 (145.36)	0.662
	PP	57	477.65 (222.47)	45	451.09 (53.95)	0.474

Abbreviations: *ITT* intention-to-treat analysis, *PP* per-protocol analysis.

*Mann–Whitney *U* test.

Education and self-care tools were delivered differently to each group. The CG had support during in-person sessions, but no data was collected regarding the educational component. The DG read a median of 4.0 (IQR 6.0) educational articles and engaged with 6.0 (IQR 7.3) cognitive behavioral therapy (CBT) content pieces. Additionally, these participants communicated with the PT through a mean of 2.8 (SD 3.0) video and phone calls and 19.0 (SD 11.8) text conversations across the program. Satisfaction with the program was high and similar between groups (*P* = 0.837): DG: 8.4/10 (SD 2.0) and CG: 8.4/10 (SD 2.6).

### Clinical outcomes

The results following an ITT analysis are presented in Table 3, while the per-protocol analysis is in Supplementary Table 3.

**Table 3:** Outcomes changes between baseline and 8 weeks: ITT analysis (*N* = 140).

Outcome Variables (median; 95% CI)	*N*	Digital group	*N*	Conventional group	Estimate the difference between groups	P*
*ODI*						
Baseline	70	24.84 (19.93; 28.75)	70	25.34 (21.91; 28.77)	−0.50 (−4.91; 3.91)	0.821
8 weeks		17.94 (6.37; 19.72)		18.99 (15.20; 22.78)	−1.05 (−4.14; 6.37)	0.671
Change baseline-8 weeks		−6.90 (−19.33; −1.57)		−6.35 (−10.36; −3.83)	−0.55 (−2.42; 5.81)	0.412
*Pain level*						
Baseline	70	5.40 (4.59; 6.02)	70	5.49 (5.05; 5.94)	−0.09 (−0.81; 0.62)	0.795
8 weeks		3.59 (0.86; 2.71)		3.38 (2.71; 4.05)	0.21 (−0.76; 0.84)	0.913
Change baseline-8 weeks		−1.81 (−3.51; −0.25)		−2.11 (−2.82; −1.49)	0.30 (−0.71; 1.10)	0.666
*Surgery intent*						
Baseline	70	6.43 (0.00; 20.68)^a^	70	9.76 (4.88; 14.64)	−3.32 (−11.37; 4.72)	0.410
8 weeks		0.06 (0.00; 1.49)^a^		0.06 (0.00; 4.77)^a^	0.00 (−10.30; 3.92)	0.372
Change baseline-8 weeks		−6.37 (−25.06; −2.92)		−9.69 (−13.60; −4.38)	3.32 (−3.11; 5.90)	0.788
*FABQ-PA*						
Baseline	70	14.41(12.06; 14.78)	70	13.99 (12.50; 15.48)	0.42 (−1.43; 2.27)	0.650
8 weeks		12.48 (8.41; 12.71)		12.48 (10.65; 14.32)	0.00 (−1.55; 1.69)	0.928
Change baseline-8 weeks		−1.92 (−5.48; 2.48)		−1.50 (−3.28; 0.03)	−0.42 (−2.10; 1.78)	0.871
*GAD-7*						
Baseline	70	3.79 (1.32; 5.88)	70	4.22 (2.92; 5.51)	−0.43 (−1.87; 1.02)	0.555
8 weeks		2.51 (0.00; 2.72)^a^		2.51 (1.36; 3.67)	0.00 (−0.99; 1.21)	0.842
Change baseline-8 weeks		−1.28 (−4.35; −0.91)		−1.70 (−2.16; −0.30)	0.43 (−0.59; 1.59)	0.360
*PHQ-9*						
Baseline	70	4.09 (0.45; 4.87)	70	4.67 (3.54; 5.80)	−0.58 (−2.13; 0.97)	0.454
8 weeks		3.22 (1.05; 3.67)		2.48 (1.44; 3.52)	0.74 (−0.53; 1.68)	0.302
Change baseline-8 weeks		−0.87 (−4.43; −0.51)		−2.19 (−2.86; −1.14)	1.33 (−2.20; 2.46)	0.095
*WPAI overall*						
Baseline	45	25.61 (13.81; 30.80)	45	23.79 (18.84; 28.74)	1.82 (−4.37; 8.02)	0.557
8 weeks		16.87 (0.00; 18.98)^a^		17.67 (10.29; 25.05)	−0.80 (−12.12; 2.70)	0.208
Change baseline-8 weeks		−8.74 (−25.98; −2.72)		−6.12 (−9.25; 4.97)	−2.62 (−14.87; 4.80)	0.122
*WPAI work*						
Baseline	43	23.52 (11.32; 29.49)	44	22.51 (17.62; 27.40)	1.01 (−5.04; 7.07)	0.738
8 weeks		15.33 (0.00; 17.62)^a^		16.29 (8.86; 23.73)	−0.96 (−10.39; 1.47)	0.137
Change baseline-8 weeks		−8.19 (−22.87; 0.03)		−6.22 (−10.14; 2.68)	−1.97 (−12.69; 3.33)	0.246
*WPAI time*						
Baseline	43	3.35 (0.00; 13.08)^a^	44	2.63 (0.29; 4.96)	0.73 (−2.31; 3.76)	0.633
8 weeks		0.00 (0.00; 13.08)^a^		0.00 (0.00; 2.34)^a^	0.00 (−5.14; 1.80)	0.338
Change baseline-8 weeks		−3.36 (−9.01; −1.51)		−2.63 (−4.38; −1.99)	−0.73 (−6.50; 2.69)	0.408
*WPAI activity*						
Baseline	70	35.59 (27.39; 44.10)	70	36.73 (29.60; 43.87)	−1.14 (−10.42; 8.13)	0.806
8 weeks		22.66 (1.11; 28.34)		23.45 (17.22; 29.69)	−0.80 (−7.43; 9.28)	0.825
Change baseline-8 weeks		−12.93 (−32.09; −3.11)		−13.28 (−19.27; −7.38)	0.35 (−6.22; 9.87)	0.650

Abbreviations: *FABQ-PA* fear-avoidance beliefs questionnaire for physical activity, *GAD-7* generalized anxiety disorder 7-item scale, *ODI* Oswestry Disability Index, *PHQ-9* Patient Health 9-item questionnaire, *WPAI* work productivity and activity impairment questionnaire.

*Quantile mixed-effects model using a robust method on the medians.

^a^Confidence intervals were fixed to zero because the analysis provided results outside the range of the corresponding scale.

Both intervention groups started with similar baseline *Oswestry Disability Index (ODI)* scores (median difference: −0.50, 95%CI −4.91;3.91; *P* = 0.821) and reported significant within-group improvements at program-end (DG: −6.90, 95% CI −19.33 to −1.57 and CG: −6.35, 95% CI: −10.36 to −3.83, both *P* < .001; Table 3). Changes were not significantly different between groups (median difference: −0.55, 95% CI: −2.42 to 5.81, *P* = 0.412), corresponding to an effect size of −0.13. The 8-week-end scores also did not significantly differ between groups (−1.05, 95% CI: −4.14 to 6.37; *P* = 0.671, Table 3). Per-protocol analysis yielded similar results (Supplementary Table 3).

The proportion of responders at the program end was similar between groups (DG: 40.4% (23/57) and CG: 42.2% (19/45)), denoted by a non-significant odds ratio (OR) of meeting minimal clinically important difference (MCID) (OR: 0.926, 95% CI: 0.42–2.05, *P* = 0.849).

Participants from both groups reported moderate pain levels at baseline (Table 3), which improved significantly in both groups (DG: −1.81, 95% CI: −3.51 to −0.25 and CG: −2.11, 95% CI −2.82 to −1.49; both *P* < 0.001). There were no differences in pain reduction between groups at the 8-week primary endpoint (0.21, 95% CI: −0.76 to 0.84; *P* = 0.913) or overall change (0.30, 95% CI −0.71 to 1.10; *P* = 0.666), as reflected by an effect size of −0.08.

Willingness to pursue surgery was low at baseline (DG: 6.43, 95% CI 0.00–20.68; CG: 9.76, 95% CI: 4.88–14.64), with only 34.3% (24/70) of participants in the DG and 42.9% (30/70) of participants in the CG reporting surgery intent levels above zero. Nevertheless, significant improvements were observed, with both groups reporting scores close to zero at the program-end. Both end scores and overall change were not significantly different between groups (Table 3).

Among those participants taking analgesics at baseline, 9/34 participants in the DG (26.5%) and 7/43 in the CG (16.3%) reported taking opioids (*P* = 0.274). Analgesic consumption remained stable in both groups (DG: *P* = 0.515, CG *P* = 0.076) until the program-end. The OR for analgesic or opioid consumption was not statistically different between groups (analgesics OR: 0.92, 95% CI: 0.00–1.23, *P* = 0.081; opioids OR: 0.26, 95% CI: 0.00–1.71, *P* = 0.985).

A similar proportion of patients reported having at least mild anxiety (Generalized Anxiety Disorder 7-item scale—GAD-7 scores ≥5: DG: 24/70 and CG:27/70) or depression (Patient Health 9-item Questionnaire—PHQ-9 scores ≥5: DG: 29/70 and CG:32/70) symptoms at baseline. Both groups showed improvements in anxiety and depression, which were not significantly different (overall change median difference: GAD-7: 0.43, 95% CI: −0.59 to 1.59; *P* = 0.360; PHQ-9: 1.33, 95% CI −2.20 to 2.46; *P* = 0.095). Regarding fear-avoidance beliefs (FAB), both groups evolved similarly as depicted by the similar 8-week end scores and changes.

Regarding physical activity levels, only the DG experienced a statistically significant within-group change towards a higher physical activity category at 8 weeks (DG: 0.66, *z*-score −2.71; *P* = 0.008 vs. CG: 0.38, *z*-score −1.43; *P* = 0.155) with no between-groups differences observed (*P* = 0.886, Table 4).

**Table 4:** Physical activity levels of participants: ITT analysis.

Physical activity levels		Digital group	Conventional group	P*
Baseline	High	17 (29.8%)	14 (31.1%)	0.934
	Moderate	19 (33.3%)	16 (35.6%)	
	Low	21 (36.8%)	15 (33.3%)	
8-weeks	High	23 (40.4%)	17 (37.8%)	0.886
	Moderate	20 (35.1%)	15 (33.3%)	
	Low	14 (24.6%)	13 (28.9%)	

*Ordinal regression.

Very low levels of absenteeism were observed in both groups, while presenteeism, overall productivity, and non-work-related activities impairments were more expressive at baseline. Significant improvements were observed in work productivity and activity impairment (WPAI) overall, WPAI work, and non-work-related activity impairment in both groups. No differences were observed in recovery or 8-week-end scores between groups (Table 3). Absenteeism levels remained stable in both groups.

### Subgroup analysis—lumbar radicular pain

Both groups comprised a similar proportion of patients with radicular LBP at baseline (DG: 21/70 (30.0%) and CG: 23/70 (32.9%), *P* = 0.716; Table 1).

Comparing those presenting with and without radicular pain, no differences were found in any baseline clinical score, considering both the entire cohort and individually in each group (all *P* > 0.05, data not shown). At the program-end, no differences were found in 8-week scores for any clinical outcome (all *P* > 0.05, data not shown).

### Safety and adverse events

No differences were observed in adverse event rates between groups (DG: 17.5% versus CG: 10.5%, *P* = 0.277). No serious adverse events or hospitalizations were reported, and none of the adverse events were related to the programs (Supplementary Table 4).

## Discussion

This study provides evidence of the effectiveness of a digital asynchronous intervention compared to high-intensity in-person physiotherapy for CLBP. High program adherence was found in both groups, with similar treatment dosages. Dropout rates were lower in the DG. Both groups achieved significant improvements in disability and pain with no statistical differences in changes or 8-week-end scores. The proportion of treatment responders was also similar between groups (DG: 40.4% and CG: 42.2%; *P* = 0.849). Additionally, no significant differences between groups were found for secondary outcomes.

The comparable improvements between both rehabilitation modes are of particular importance since they illustrate the effectiveness of digital care. The digital format favors the democratization of healthcare access by overcoming geographical barriers (reaching rural or underserved areas), decreasing treatment start waiting time, and promoting patient engagement while ensuring the quality of care and potentially lowering costs. These results demonstrate that a fully-remote digital intervention for CLBP can promote similar improvements as high-intensity evidence-based in-person physiotherapy.

We observed a high enrollment, with only 12.7% of all screened individuals declining to participate, and a low dropout rate (DG: 15.7% and CG: 34.3%). This enhances generalization and supports the increased acceptance of remote care, in which the COVID-19 pandemic played a role. Similar high satisfaction levels were reported by patients from both groups, further reinforcing participants' acceptance, consistent with previous findings^17^.

Compliance with physiotherapy is known to be challenging^13^, even in the setting of RCTs^26^. Digital interventions have been reported to have similar^16,20,27^ or lower^21^ dropout rates than in-person physiotherapy. In the present study, the DG had lower dropout rates compared to the CG, potentially explained by the DCP convenience, reducing travel and treatment time hurdles and allowing greater flexibility on patient’s schedules, as previously reported^17,28^. Additionally, the context during the COVID-19 pandemic may have impacted receptivity, compliance, and overall perceptions regarding digital programs.

High engagement was observed in both groups, which translated into similar treatment dosages, as intended through the study design. The drivers for engagement with in-person physiotherapy have long been the focus of research^29^. In the DG, the high engagement noted might be the result of remote monitoring capabilities, which have been reported to improve self-efficacy and motivation^17,28^, and through bi-directional communication between patients and doctors of physiotherapy (DPTs) (including video and phone calls and asynchronously through messages)^30^. Frequent communication is a critical component in establishing a therapeutic alliance, a well-known factor associated with better outcomes^31^. The interest denoted by participants in the DG in continuing the program after the study end (data not shown) highlights the convenience of this care modality.

The specific optimal dosage of exercise (i.e., frequency and time) in CLBP rehabilitation is still unknown; therefore, recommendations range widely and are ideally personalized (2–5 times per week; 20–60 min)^32^. The treatment dosage reached by participants in this study falls within the recommended range.

Both groups improved similarly in disability and pain, denoted by small effect sizes (ODI: −0.13; Pain: −0.08). These results are consistent with previous trials comparing exercise-based telerehabilitation to in-person rehabilitation for patients with LBP with diverse acuity^16,20,21,27^, which found similar outcomes in disability and pain. These results indicate that digital interventions yield similar results to those achieved with in-person care, even in chronic conditions.

Co-prevalence of pain and mental distress is very common among chronic musculoskeletal conditions^33^ due to a strong and complex bidirectional relationship^34^, contributing to a poorer prognosis^35^. Exercise-based telerehabilitation interventions can positively impact mental health across several conditions^24,36^, including CLBP^25,37^. In this study, both groups experienced significant improvements in anxiety and depression, with no significant differences between groups. Likewise, FABs have been associated with non-recovery^38^. Herein, both groups started with comparable baseline scores and reported similar improvements, supporting that physiotherapy helps to overcome movement avoidance due to fear of getting worse^38,39^. This shift in attitude, together with disability and pain improvements, may have contributed to the significantly increased levels of physical activity observed in the DG at the program-end. Indeed, strategies that promote healthy lifestyle habits, including physical activity, have long been recommended for CLBP^9,10^.

Work productivity is greatly affected by CLBP^40^, as observed in this cohort, with most participants reporting some level of absenteeism or presenteeism. It is known that physiotherapy can decrease absenteeism and presenteeism^2,41^. Herein, absenteeism was low at baseline and remained stable until program-end in both groups. However, absenteeism levels may be influenced by factors other than condition severity, such as financial support, work culture, secondary gain, coping skills, job satisfaction, and the ability to modify work^42^. Improvements in presenteeism were observed in both interventions without significant differences between groups.

Non-work-related activities can also be compromised in those suffering from CLBP^43^. In this study, both groups reported improvements in these activities, with no significant differences between them at the program-end, in line with current knowledge that physical activity can improve one’s ability to complete activities of daily living^43^.

Non-surgical treatments for CLBP have shown comparable or better outcomes in disability and return to work than surgical interventions^2,12^. Herein, the patient’s willingness to pursue surgery at baseline was low and decreased to near zero at the intervention-end, which is likely attributable to the reduction in pain and disability. This is auspicious considering that the patient’s surgery intention is one of the strongest predictors of future surgery^44,45^ and that many spinal surgeries are unnecessary^2,5,12^.

Opioid overuse and misuse represent a substantial problem in CLBP^46^. Opioid prescription rates have been reported to be twice as high as physiotherapy referral^7^, which contrasts with clinical recommendations^9,10^. A National survey conducted in the U.S. found that 21.5% of patients with chronic musculoskeletal pain take opioids^7^, while a cross-sectional study reported that 36.9% of American adults suffering specifically from CBLP use some type of analgesics^8^. In the present study, approximately half of the participants in both groups used analgesics, but only 10–13% reported opioid consumption at baseline. Both groups maintained a similar proportion of patients consuming analgesics until program-end despite the observed decreases in pain and disability, which is similar to a previous trial reporting no differences between in-clinic and web-based rehabilitation for LBP^20^. In future studies, higher granularity in medication assessment (i.e., type, dosage, and intake frequency) would help to better assess the effect of interventions in reducing pharmacological intake. Nevertheless, early physiotherapy has been reported to lower the risk of opioid misuse^47,48^.

In the context of insufficient resources and increasing demand for rehabilitation services^1^, alternative and scalable care delivery systems are needed. The growing body of evidence around digital care supports their use to overcome existing gaps^15–17^. These models will pave the way for a new integrated approach featuring digital, in-person, and hybrid care models. This will have profound cultural, clinical, and organizational implications for healthcare providers. Technology can be used to expand reach, promote timely access to care and provide critical data on patient progress and outcomes, but it requires appropriate candidate selection. Further research is required to determine the best candidates for each approach (digital, in-person or hybrid), considering clinical and demographic characteristics, and patient preference, as evidenced by changing attitudes towards digital care during the pandemic^49^. The widespread adoption of these new models requires the acquisition of new competencies by physiotherapists, requiring important changes in training curricula. Additionally, studies with long-term follow-ups and cost-effectiveness analyses are warranted.

A key strength of this study is the methodology — an RCT comparing a full DCP against high-intensity, in-person physiotherapy, with balanced treatment dosages between both groups. Of note, the treatment dosages used in both groups were much higher than typically utilized in U.S. outpatient clinics^50^. Other aspects of methodological rigor included randomization and allocation concealment, clinically relevant secondary outcomes, ITT analysis, reporting according to CONSORT guidelines, and trial registration. The novelty of the digital intervention is another strength of this study — a fully-remote multimodal DCP, asynchronously managed by a DPT, combining exercise with real-time biofeedback to education and CBT. The use of biofeedback guiding patients during sessions^18,30^ and the close monitoring and communication with the DPT may increase patient’s adherence^17,28,29^. This study is not without limitations, which include the lack of (i) blinding of patients, DPTs and investigators, due to the nature of the interventions; (ii) objective outcome measures (e.g., muscle strength, range of motion), including a metric for physical activity; and (iii) an externally blinded outcome adjudicator. Second, the study was conducted during and post-COVID-19 pandemic, which may have positively impacted compliance rates for both groups, more so in the digital intervention. Third, given the higher dropout rate in the CG, additional strategies to increase motivation and engagement could have been implemented, such as additional contacts between in-person sessions. Fourth, more granularity for the pharmacological variable (e.g., type, dosage, or frequency of analgesic intake) should be addressed in future studies. Finally, interventions performed under controlled environments may not reflect real-world conditions despite liberal selection criteria that yielded a population comparable to the reported in previous large-scale surveys and studies^51^.

In conclusion, this study supports the comparable effectiveness of a fully-remote digital intervention for CLBP patients compared to high-intensity, evidence-based, in-person physiotherapy. Similar improvements were noted in both groups, as well as high adherence and satisfaction, with lower dropout rates in the digital group. In the face of the high and growing burden of CLBP, these results support the consideration of digital interventions as viable and effective alternatives to in-person care, ensuring clinical quality and safety while reducing barriers to access.

## Methods

### Study design

This single-center, parallel-group, randomized controlled study was conducted in accordance with the Declaration of Helsinki. Following CONSORT guidelines, the trial was prospectively approved by the Emory Institutional Review Board (number STUDY00001546) and registered on ClinicalTrials.gov (NCT04808141) on March 22nd, 2021. Treatment occurred from June 15th, 2021, through October 26th, 2022.

### Participants

Patients seeking care at the Emory Orthopedic and Spine Center (Atlanta, Ga) were screened for eligibility in-clinic by a physician who followed up participants until the study end. Informed written electronic consent was obtained from all participants (Castor eConsent, Castor Research Inc.). All study-related data was stored in an electronic data capture system (Castor EDC, Castor Research Inc.). Inclusion criteria were: (a) subjects between 18 and 80 years of age; (b) CLBP — intermittent or persistent LBP for at least 12 weeks, and/or ≥50% of the time in the past 6 months^52^; (c) ability to understand complex motor tasks; (d) ability to interact with a tablet. Exclusion criteria were: (a) known pregnancy; (b) spinal surgery <3 months ago; (c) symptoms and/or signs indicative of infection; (d) indication for spine surgery (i.e., tumor, cauda equina syndrome); (e) cancer diagnosis or undergoing cancer treatment; (f) known disorder incompatible with 20-minute light to moderate exercise; (g) concomitant non-spine related neurological disorder (e.g., stroke and multiple sclerosis); (h) dementia or psychiatric disorders precluding a patient from complying with a home-based exercise program; (i) illiteracy and/or serious visual or auditory impairment interfering with communication or compliance. Participants presenting with lumbar radicular pain were allowed to enter the study and were identified by: pain radiating below the buttock in a dermatomal distribution, altered sensation or paresthesia (e.g., hypoesthesia, numbness, and tingling), neurologic signs, or exam findings consistent with radicular pain^53^. To enroll in the study, participants were required to stop ongoing physiotherapy for CLBP.

After eligibility screening, participants who did not complete the assessment surveys or suffered serious adverse events^54^ were excluded. Participants were considered dropouts if they: (1) abandoned the study; or (2) did not engage in any exercise session for 28 consecutive days in the DG or missed 4 consecutive scheduled sessions in the CG.

### Allocation and blinding

All participants providing consent were randomized into the DG or CG in a 1:1 ratio, using random permuted blocks of 4–8 participants, automatically generated by the Castor EDC platform. Group disclosure was only performed after randomization (concealed allocation), after which the principal investigator (D.C.) communicated the assignment to the study coordinator (L.G.). Blinding physiotherapists and patients to allocation was not possible, given the nature of the intervention.

### Interventions

The digital intervention group received an 8-week telerehabilitation intervention consisting of exercise complemented with education, and CBT delivered through a digital platform (Supplementary Table 5), which interfaced between the participant and the assigned DPT. DPTs involved in the study had, on average, 13 years of experience (range 6–23). An FDA-listed class II medical device comprising two inertial motion trackers, a mobile app on a dedicated tablet, and a cloud-based portal was made available to all participants. Hot spots were sent to participants without an internet connection. After an initial onboarding video call where the DPT assessed each participant, a tailored program was prescribed. Exercise sessions (three 20-minute sessions per week recommended^32^; a total of 24 sessions) were performed independently at the participants’ convenience through the tablet display. The system provided real-time biofeedback on performance through video-audio cues based on trackers placed with straps on the thoracic and lumbar regions (Supplementary Fig. 2).

Data on exercise session performance (range of motion, execution, and movement compensations) and engagement (number of executed or skipped repetitions, and time dedicated to exercise) was automatically recorded by the tablet app and stored in a cloud-based portal, enabling asynchronous monitoring by the assigned DPT, who performed adjustments to protocol.

The educational component consisting of articles focusing on anatomy and physiology, pain, exercise, and fear-avoidance behaviors^9,10^, was delivered through a smartphone app. A dedicated third-generation CBT program combining mindfulness, acceptance, commitment therapy, and empathy-focused therapy, adapted to a curriculum focused on chronic pain consisting of self-paced written and pre-recorded audio materials, was delivered through email^9,10^. Both education and CBT components were developed according to current guidelines^9,10^. Bi-directional communication was ensured by a built-in secure chat within a smartphone app or calls (with touchpoints scheduled every 4 weeks and on-demand), which was also intended to motivate and engage patients to the intervention. Members had access to technical and IT support through several communication channels. Hardware issues that could not be resolved remotely were solved by replacing the device.

The conventional intervention group received evidence-based in-person physiotherapy composed of exercise (following Emory’s standardized protocols: including strengthening, balance, stretching, and mobility exercises; similar to the digital group), education (regarding pain physiology, fear-avoidance behaviors and benefits/effects of exercise) on an ongoing and as-needed basis, manual therapy (e.g., joint mobilization and massage) and physical modalities (e.g., electrical stimulation) (Supplementary Table 6). DPTs involved in the study had, on average, 12 years of experience (range 7–20). The program was adjusted according to the participant’s condition and consisted of two 30-minute sessions per week for 8 weeks (total of 16 sessions)^32^. In specific circumstances, combining the two 30-minute sessions into one 60-minute session was allowed to circumvent unforeseen scheduling issues. Participants were also instructed to perform the exercises at home, but adherence to this was not assessed.

### Outcomes

Outcomes were collected at baseline, 4, and 8 weeks (except physical activity levels at baseline and 8 weeks). Changes were calculated between baseline and 8 weeks.

The primary outcome was the Oswestry Disability Index (ODI) change between baseline and 8 weeks. ODI is validated for CLBP (with and without radiculopathy)^55^, including 10 items with a 6-point Likert scale (score: 0 (none)–100% (worse))^56^. The designation of a treatment responder was considered to be an MCID of 10-points or 30%^57^ at the primary 8-week endpoint. Secondary outcomes are presented in Table 5.

**Table 5:** Study secondary outcomes (clinical and engagement).

Outcome measure	Description
Self-reported pain level	An 11-point Numerical Pain Rating Scale (NPRS) for average pain in the last 7 days (0: no pain; 10: worst pain imaginable)^63^
Intention to undergo surgery	“On a scale of 0 to 100, where 0 is not at all, and 100 is extremely interested, how interested are you in undergoing shoulder surgery in the next 12 months?”
Analgesics consumption	“Are you taking any medication for your shoulder pain? Yes/No”; and opioid consumption “If yes, are you taking opioids for your low back pain? Yes/No”. Although further information regarding opioid dosage was intended to be collected, this data was not systematically recorded across the study
Fear-Avoidance Beliefs	Measured through the 5-item FAB questionnaire for physical activity (FABQ-PA; range 0–24)^64^
Mental Health	Generalized Anxiety Disorder 7-item scale (GAD-7) (range 0–21)^65,66^ to assess anxiety, and Patient Health 9-item questionnaire (PHQ-9) (range 0–27) to assess depression^66,67^
Physical Activity	International Physical Activity Questionnaire - Short Form (IPAQ)^68^, including 7 items focusing on physical activity. Scoring and categorization (low, moderate, and high) followed guidelines established by the IPAQ Research Committee^69^
Work Productivity Impairment	Work Productivity and Activity Impairment (WPAI) for General Health questionnaire (version 2.0), applied to employed participants to assess overall work impairment (WPAI overall: presenteeism and absenteeism), presenteeism (WPAI work) and absenteeism (WPAI time). Non-work-related activity impairment (WPAI activity) was assessed in all participants. Higher scores denote greater impairment^70^
Engagement	assessed through (i) adherence to exercise sessions (measured as the number of attended versus scheduled sessions); (ii) dropout rates; (iii) treatment dosage (total time spent on exercise sessions in minutes); automatically collected by the tablet app in the DG and manually recorded by DPT in the CG. Additionally, the number of educational and CBT content pieces consulted, and the number of contacts between DPT and patients were automatically collected by the mobile app or email in the DG or manually recorded by DPT in the CG.
Patient satisfaction	“On a scale from 0 to 10, how likely is it that you would recommend this intervention to a friend or neighbor?”.

### Safety and adverse events

CG sessions were performed under the direct supervision of a DPT. In the DG, exercise performance (motion trackers-based), as well as pain and fatigue data (faces-rating 5-item scale), were obtained following each session to support the PT asynchronous monitoring. Both participants and DPTs were instructed to contact the study investigators when adverse events occurred (registered on Castor EDC).

### Sample size

The sample size estimation was based on ODI (primary outcome). Considering the literature at the time of protocol submission, a standard deviation at a baseline of 17.83 was applied, supported by the study of Stankovic et al.^58^, who compared two different rehabilitation protocols for CLBP (*N* = 160). A difference between groups of 10-points was considered to be clinically meaningful based on the study by Ostelo et al.^57^. Considering a power of 80% and a two-sided 0.05 significance level, we calculated that 102 individuals would be necessary to detect a 10-point difference between the two groups. To guarantee that the study was adequately powered to detect equivalence, a posteriori analysis was conducted using the Two One-Sided Test (TOST) methodology^59^ (simulation-based power analysis). Assuming an 80% power to detect equivalence, a total of 104 participants (52 per arm) was estimated. A dropout rate of 15% was considered, which required to enroll 120 participants (60 per arm). To assess non-inferiority, a smaller sample size would be needed (total of 74; 37 per arm). To account for screening failures and dropouts, the participant enrollment period was extended beyond the original target.

### Statistical analysis

Continuous data distribution was analyzed using the Kolmogorov–Smirnov test, followed by inspection of histograms and Q-Q plots. Baseline demographic and engagement metrics differences between groups were assessed using independent samples *t*-test or Mann–Whitney *U* test for quantitative variables and the Chi-square test for categorical variables. Baseline medication, opioid consumption, and the occurrence of adverse events at 8 weeks between groups were assessed through the Chi-square test.

The impact of each intervention on the primary and secondary outcomes was assessed using both the 8-week end scores and changes between baseline and 8 weeks. Outcomes were analyzed following both ITT, considering all randomized participants, and per-protocol analyses.

Since the assumption of normality was not met (Supplementary Fig. 1), logarithmic and Box–Cox transformations were performed. Results indicated that these transformations were not able to achieve normality (data not shown), and therefore, the repeated measures ANOVA analysis planned in the protocol was not conducted. As an alternative, a quantile mixed-effects model using a robust method on the medians was performed for the ITT analysis. This previously validated model accounts for repeated measurements and assumes non-normality^60^. Missing data were handled using multiple imputations by chained equations (MICE)^61^.

Considering group sizes in the per-protocol analysis (precluding quantile mix-effects model analysis), differences between interventions were evaluated using Mann–Whitney *U* tests with the Hodges–Lehmann estimator.

Considering the poorer prognosis associated with untreated radicular pain^62^, which affects care decision-making^9,10,53^, a subgroup analysis comparing clinical outcomes between those presenting with lumbar radicular pain and those without radiculopathy was performed using Mann–Whitney *U* tests.

Binary logistic regression analysis was performed to identify the odds of being a responder for ODI, considering a 10-points or 30% MCID^57^. This analysis was also conducted to assess the odds between groups for consuming analgesics, including opioids, at 8 weeks using the CG as a reference.

Physical activity was assessed using ordinal regression to evaluate whether the latent distribution of physical activity categories changed significantly from baseline to program-end within and between groups.

In all analyses, a two-sided hypothesis test with an alpha level of 0.05 was considered statistically significant. Robust linear mixed effects model and ordinal regression were coded using R (version 4.2.2, R Foundation for Statistical Computing) and all other analyses using SPSS (version 28.0, SPSS Inc, Chicago, Illinois, USA). Analysis of the data was performed by a blinded statistician.

## Supplementary information

Supplementary Information

## Data Availability

The study will be available in clinicaltrials.gov upon publication for at least 5 years. Aggregated data of this study’s findings will be available upon reasonable request from the corresponding author.

## Code availability

Code for all statistical analyses may be available upon reasonable request from the corresponding author.
