# Supplementary material for: Randomized-controlled trial assessing a digital care program versus conventional physiotherapy for chronic low back pain
Source: NPJ Digit Med. 2023 Jul 7;6:121. doi: 10.1038/s41746-023-00870-3 (PMC10329005; doi:10.1038/s41746-023-00870-3)
Supplement: Supplementary file 1 — Supplementary Information [file 41746_2023_870_MOESM1_ESM.pdf]

SUPPLEMENTARY INFORMATION

Supplementary Figure 1. Histograms and normality tests to assess data distribution for disability (Oswestry Disability Index - ODI) and pain levels.

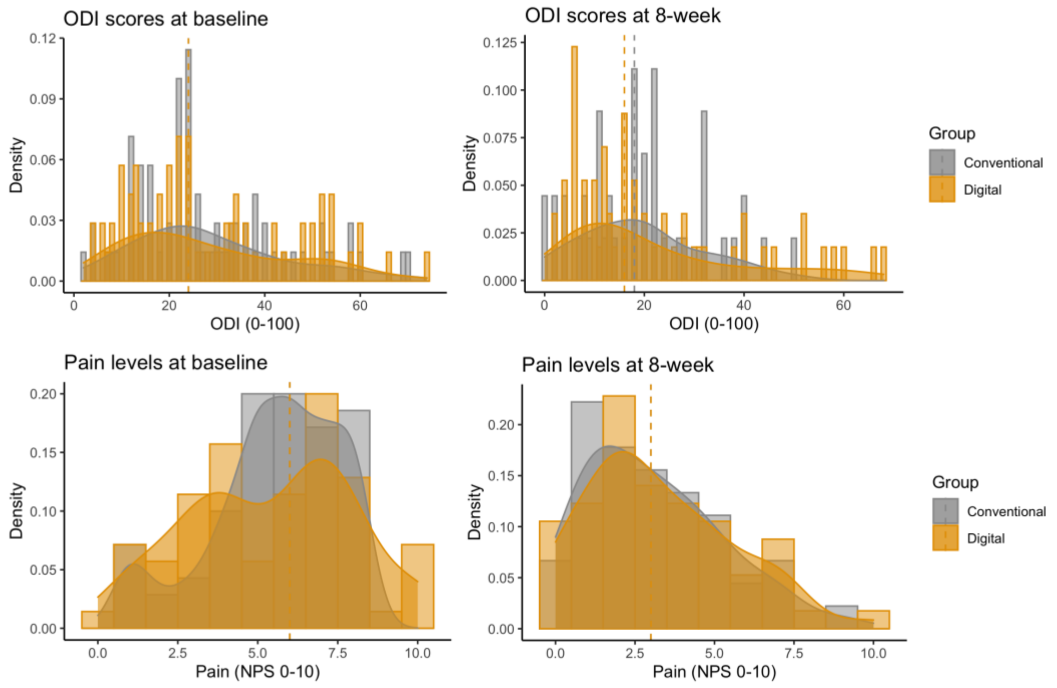

Tests of Normality

	Group	Kolmogorov-Smirnov[a] Statistic	df	Sig.	Shapiro-Wilk Statistic	df	Sig.
ODI scores at baseline	Conventional	.079	45	.200*	.976	45	.470
	Digital	.139	57	.008	.927	57	.002
ODI scores at 8-week	Conventional	.120	45	.109	.965	45	.194
	Digital	.184	57	<.001	.863	57	<.001

*. This is a lower bound of the true significance.
a. Lilliefors Significance Correction

Tests of Normality

	Group	Kolmogorov-Smirnov[a] Statistic	df	Sig.	Shapiro-Wilk Statistic	df	Sig.
Pain levels at baseline	Conventional	.166	45	.003	.915	45	.003
	Digital	.152	57	.002	.959	57	.052
Pain levels at 8-week	Conventional	.157	45	.007	.932	45	.011
	Digital	.163	57	<.001	.939	57	.006

a. Lilliefors Significance Correction

Supplementary Table 1. Baseline characteristics of study completers.

Characteristic	Digital Group (N=57)	Conventional Group (N=45)	P*
Age (years), median (IQR)	47.00 (24.00)	59.00 (19.00)	**.040**
Age categories, N (%):			**.039**
<25	1 (1.8)	1 (2.2)	
25-40	19 (33.3)	5 (11.1)	
41-60	24 (42.1)	20 (44.4)	
> 60	13 (22.8)	19 (42.2)	
Gender, N (%):			.363
Woman	37 (64.9)	33 (73.3)	
Man	20 (35.1)	12 (26.7)	
BMI, median (IQR)	28.06 (9.23)	28.48 (9.26)	.793
BMI categories, N (%):			.928
Normal (18.5-25)	20 (35.1)	16 (35.6)	
Overweight (>25-30)	17 (29.8)	11 (24.4)	
Obese (>30-40)	16 (28.1)	14 (31.1)	
Morbidly obese (>40)	4 (7.0)	4 (8.9)	
Race, N (%):			.231
Asian or Pacific Islander	10 (17.5)	5 (11.1)	
Black or African American	18 (31.6)	21 (46.7)	
Hispanic or Latino	3 (5.3)	1 (2.2)	
Native American or Alaskan Native	0 (0.0)	1 (2.2)	
White or Caucasian	25 (43.9)	15 (33.3)	
Multi-racial or biracial	0 (0.0)	2 (4.4)	
Prefer not to answer	1 (1.8)	0 (0.0)	
Education level, N (%):			.816
Not attended school	0 (0.0)	0 (0.0)	
Some high school	2 (3.5)	0 (0.0)	
High school graduate or GED	3 (5.3)	3 (6.7)	
Some college (some community college, associate degree)	13 (22.8)	9 (20.0)	
Four-year college degree or bachelor’s degree	15 (26.3)	12 (26.7)	
Some post-graduate or professional schooling, no postgraduate degree	4 (7.0)	5 (11.1)	
Postgraduate or professional degree (including master’s, doctorate, medical or law degree)	20 (35.1)	16 (35.6)	
Prefer not to answer	0 (0.0)	0 (0.0)	
Employment status, N (%):			.497
Employed (part-time or full-time)	38 (66.7)	30 (66.7)	
Unemployed (seeking opportunities)	7 (12.3)	2 (4.4)	
Not employed	11 (19.3)	12 (26.7)	
Prefer not to answer	1 (1.8)	1 (2.2)	
Exercise levels, N (%):			.152
None	5 (8.8)	7 (15.6)	
Less than 1 hour	18 (31.6)	7 (15.6)	

Between 1-2.5 hours	16 (28.1)	19 (42.2)	
>2.5 hours	18 (31.6)	12 (26.7)	
Comorbidities, N (%):			
High blood pressure	18 (31.6)	15 (33.3)	.851
High blood sugar or diabetes	6 (10.5)	4 (8.9)	.782
Cardiac conditions	3 (5.3)	1 (2.2)	.432
Respiratory conditions	8 (14.0)	7 (15.6)	.830
None of the listed	32 (56.1)	24 (53.3)	.777
Smoking habits, N (%):	1 (1.8)	0 (0.0)	.372
Low back-related leg pain, N (%):	28 (49.1)	26 (57.8)	.385
Laterality of leg pain, N (%):			.884
Right	10 (35.7)	11 (42.3)	
Left	12 (42.9)	10 (38.5)	
Both	6 (21.4)	5 (19.2)	
Lumbar radicular pain, N (%):	18 (31.6)	18 (40.0)	.377
Previous physiotherapy, N (%):	30 (52.6)	25 (55.6)	.769
Previous or scheduled low back surgery, N (%):	5 (8.8)	3 (6.7)	.695

Abbreviations: BMI, Body mass index; GED, General Educational Development (includes technical or vocational training).

*Mann–Whitney U test or Pearson Chi-Square; Note: Significant p-values are presented in bold.

Supplementary Table 2. Baseline characteristics between study completers (N=102) and non-completers (N=38).

Characteristic	Completers (N=102)	Non-completers (N=38)	P*
Age (years), median (IQR)	52.09 (14.27)	50.50 (14.74)	.563[§]
Age categories, N (%):			.373
<25	2 (2.0)	3 (7.9)	
25-40	24 (23.5)	7 (18.4)	
41-60	44 (43.1)	17 /44.7)	
> 60	32 (31.4)	11 (28.9)	
Gender, N (%):			.749
Woman	70 (68.6)	25 (65.8)	
Man	32 (31.4)	13 (34.2)	
BMI, median (IQR)	28.22 (8.98)	29.03 (7.74)	.383[#]
BMI categories, N (%):			
Normal (18.5-25)	36 (35.3)	6 (15.8)	.151
Overweight (>25-30)	28 (27.5)	15 (39.5)	
Obese (>30-40)	30 (29.4)	14 (36.8)	
Morbidly obese (>40)	8 (7.8)	3 (7.9)	
Race, N (%):			.179
Asian or Pacific Islander	15 (14.7)	2 (5.3)	
Black or African American	39 (38.2)	22 (57.9)	
Hispanic or Latino	4 (3.9)	1 (2.6)	
Native American or Alaskan Native	1 (1.0)	0 (0.0)	
White or Caucasian	40 (39.2)	11 (28.9)	
Multi-racial or biracial	2 (2.0)	0 (0.0)	
Prefer not to answer	0 (0.0)	2 (5.3)	
Education level, N (%):			**.012**
Not attended school	0 (0.0)	1 (2.6)	
Some high school	2 (2.0)	2 (5.3)	
High school graduate or GED	6 (5.9)	6 (15.8)	
Some college (some community college, associate degree)	22 (21.6)	14 (36.8)	
Four-year college degree or bachelor’s degree	27 (26.5)	4 (10.5)	
Some post-graduate or professional schooling, no postgraduate degree	9 (8.8)	1 (2.6)	
Postgraduate or professional degree (including master’s, doctorate, medical or law degree)	36 (35.3)	9 (23.7)	
Prefer not to answer	0 (0.0)	1 (2.6)	
Employment status, N (%):			.912
Employed (part-time or full-time)	68 (66.7)	26 (68.4)	
Unemployed (seeking opportunities)	9 (8.8)	2 (5.3)	
Not employed	23 (22.5)	9 (23.7)	
Prefer not to answer	2 (2.0)	1 (2.6)	

Exercise levels, N (%):			**0.032**
None	12 (11.8)	12 (31.6)	
Less than 1 hour	25 (24.5)	8 (21.1)	
Between 1-2.5 hours	35 (34.3)	7 (18.4)	
>2.5 hours	30 (29.4)	11 (28.9)	
Comorbidities, N (%):			
High blood pressure	33 (32.4)	14 (36.8)	.617
High blood sugar or diabetes	10 (9.8)	6 (15.8)	.322
Cardiac conditions	4 (3.9)	3 (7.9)	.337
Respiratory conditions	15 (14.7)	5 (13.2)	.816
None of the listed	56 (54.9)	19 (50.0)	.605
Smoking habits, N (%):	1 (1.0)	3 (7.9)	**.029**
Low back-related leg pain, N (%):	54 (52.9)	21 (55.3)	.806
Laterality of leg pain, N (%):			.214
Right	21 (38.9)	8 (38.1)	
Left	22 (40.7)	5 (23.8)	
Both	11 (20.4)	8 (38.1)	
Lumbar radicular pain, N (%):	36 (35.3)	8 (21.1)	.106
Previous physiotherapy, N (%):	55 (53.9)	20 (52.6)	.892
Previous or scheduled low back surgery, N (%):	8 (7.8)	7 (18.4)	.072

Abbreviations: BMI, Body mass index; GED, General Educational Development (includes technical or vocational training).

*Pearson Chi-Square; §Independent samples t-test; #Mann–Whitney U test

Note: Significant p-values are presented in bold.

Supplementary Table 3. Outcomes changes between baseline and 8-weeks: per-protocol analysis (N=102).

Outcome Variables	N	Digital Group	N	Conventional Group	Estimate difference between groups§	P*
ODI						
Baseline	57	24.0 (23.7)	45	24.0 (18.9)	-0.67 (-8.00; 4.00)	.762
8 weeks	57	16.0 (22.6)	45	18.0 (16.2)	0.00 (-5.78; 5.11)	.922
Change baseline-8 weeks	57	-4.0 (8.0)	45	-2.9 (11.7)	0.00 (-3.78; 4.00)	.879
Pain Level						
Baseline	57	6.0 (4.0)	45	5.0 (3.0)	0.00 (-1.00; 1.00)	.921
8 weeks	57	3.0 (4.0)	45	3.0 (3.0)	0.00 (-1.00; 1.00)	.698
Change baseline-8 weeks	57	-2.0 (4.0)	45	-2.0 (4.0)	0.00 (-1.00; 1.00)	.641
Surgery Intent						
Baseline	57	0.0 (4.0)	45	0.0 (8.0)	0.00 (0.00; 0.00)	.717
8 weeks	57	0.0 (3.0)	45	0.0 (1.0)	0.00 (0.00; 0.00)	.222
Change baseline-8 weeks	57	0.0 (1.0)	45	0.0 (0.0)	0.00 (0.00; 0.00)	.375
FABQ-PA						
Baseline	57	15.0 (5.0)	45	13.00 (9.00)	-1.00 (-4.00; 1.00)	.395
8 weeks	57	11.0 (7.0)	45	13.0 (7.0)	-1.00 (-4.00; 1.00)	.198
Change baseline-8 weeks	57	-1.0 (7.0)	45	-2.0 (6.0)	0.00 (-2.00; 2.00)	.774
GAD-7						
Baseline	57	4.0 (6.0)	45	4.0 (6.0)	0.00 (-2.00; 1.00)	.708
8 weeks	57	3.0 (6.0)	45	2.0 (4.0)	0.00 (-2.00; 0.00)	.324
Change baseline-8 weeks	57	0.0 (3.0)	45	-1.0 (3.0)	0.00 (-1.00; 1.00)	.472
PHQ-9						
Baseline	57	4.0 (5.0)	45	3.0 (6.0)	0.00 (-2.00; 1.00)	.564
8 weeks	57	3.0 (5.0)	45	2.0 (5.0)	0.00 (-2.00; 0.00)	.242
Change baseline-8 weeks	57	0.0 (3.0)	45	-1.0 (4.0)	0.00 (-2.00; 1.00)	.344
WPAI Overall						
Baseline	36	20.0 (25.2)	32	20.0 (38.6)	0.00 (-10.00; 9.72)	.478
8 weeks	35	10.0 (20.0)	31	0.0 (36.4)	0.00 (0.00; 10.00)	.605
Change baseline-8 weeks	32	-10.0 (29.3)	31	0.0 (30.0)	10.00 (0.00; 20.00)	.112
WPAI Work						
Baseline	35	20.0 (20.0)	31	20.0 (40.0)	0.00 (-10.00; 10.00)	.656
8 weeks	35	10.0 (20.0)	30	0.0 (30.0)	0.00 (0.00; 10.00)	.655
Change baseline-8 weeks	31	-10.0 (20.0)	29	0.0 (25.0)	10.00 (0.00; 1.00)	.171
WPAI Time						
Baseline	35	0 (0)	31	0 (0)	0.00 (0.00; 0.00)	.419
8 weeks	35	0 (0)	31	0 (0)	0.00 (0.00; 0.00)	.487
Change baseline-8 weeks	31	0 (0)	31	0 (0)	0.00 (0.00; 0.00)	.488
WPAI Activity						
Baseline	57	40.0 (40.0)	45	30.0 (40.0)	0.00 (-10.00; 10.00)	.391
8 weeks	57	20.0 (30.0)	45	20.0 (40.0)	0.00 (-10.00; 10.00)	.795
Change baseline-8 weeks	57	-10.0 (25.0)	45	-10.0 (30.0)	0.00 (-10.00; 10.00)	.715

Abbreviations: FABQ-PA, Fear-avoidance beliefs questionnaire for physical activity; GAD-7, Generalized Anxiety Disorder 7-item scale; ODI, Oswestry Disability Index; PHQ-9, Patient Health 9-item questionnaire; WPAI, Work Productivity and Activity Impairment Questionnaire.

*Mann-Whitney U test; §Hodges-Lehman estimator.

Supplementary Table 4. Adverse events.

Adverse Event	Start Date	Stop date	Severity	Relationship to Study Intervention	Action taken with study treatment	Outcome of AE	Expected	Serious
Digital Group								
Brain bleeding	20/10/2021	15/12/2021	Severe	Not related	None	Unknown	No	No
Spine Procedure - lumbar medial branch block	15/10/2021	-	Moderate	Unlikely related	Other	Ongoing/Continuing treatment	No	No
Development of chest pain with visit to the emergency room	11/11/2021	15/11/2021	Moderate	Not related	None	Resolved	No	No
Fall (pain prevented walking)	13/01/2022	20/01/2022	Mild	Unlikely related	Intervention discontinued	Ongoing/Continuing treatment	No	No
Knee pain (no concrete cause)	25/11/2021	29/11/2021	Moderate	Unlikely related	None	Resolved	No	No
COVID-19 infection	25/12/2021	19/01/2022	Mild	Not related	Change of treatment program	Resolved	No	No
Low back worsening pain	23/01/2022	-	Mild	Unlikely related	Change of treatment program	Resolved	No	No
Arm cyst (urgent care needed)	20/01/2022	-	Mild	Not related	Other	Ongoing/Continuing treatment	No	No
Foot swelling	04/03/2022	-	Mild	Not related	Other	Resolved	No	No
Lumbar spinal injection reaction	01/07/2022	11/07/2022	Moderate	Not related	Change of treatment program	Resolved	No	No

Fall (injured upper extremity)	28/07/2022	-	Mild	Unlikely related	Change of treatment program	Ongoing/Continuing treatment	No	No
Conventional Group								
Ankle sprain	09/07/2021	-	Mild	Not related	None	Unknown	No	No
Low back worsening pain	03/09/2021	-	Moderate	Unlikely related	Other	Ongoing/Continuing treatment	No	No
Struck by car	12/02/2022	-	Moderate	Not related	Change of treatment program	Resolved	No	No
Fall	12/02/2022	-	Mild	Not related	None	Resolved	No	No
Cold sickness	18/01/2022	31/01/2022	Mild	Not related	None	Resolved	No	No
Low back injury after moving	30/07/2022	-	Moderate	Not related	None	Resolved	No	No

.

Supplementary Table 5. Description of the exercise prescription in the digital group.

Phases and Main goals	Intervention description
Initial Phase<br><br>Goals:<br>● Decrease symptoms intensity/severity and inflammatory signs<br>● Gradual exposure to avoided movements<br>● Increase trust in movement<br>● Improve range of motion (passive and active)<br>● Improve muscular strength and endurance<br>● Improve motor control and stability<br>● Provide knowledge on the condition, contributing factors and the role of exercise	Aim for at least 1-2 sets of:<br><br>● Articular mobility exercises (e.g. sitting trunk flexion; combined trunk flexion and rotation; lower limb and pelvic mobility)<br>● Strengthening exercises (e.g. squat, bridges)<br>● Active and passive stretching exercises (e.g. sitting posterior thigh stretch)
Intermediate Phase<br><br>Goals:<br>● Symptoms control<br>● Improve range of motion (passive and active)<br>● Increase global muscular and aerobic capacity<br>● Increase time of exposure to painful movements<br>● Gradual exposure to daily activities that elicit symptoms<br>● Integrate patient's knowledge and self-management skills into daily routine	Increase repetitions and sets or add external load (bands and/or free weights) of the prescribed exercises; Add more demanding exercises<br><br>Progressively increase average session time<br><br>Add neural glide exercises (eg. straight leg raises)<br><br>Increase exercise complexity and aim for at least 2-3 sets of:<br><br>● Articular mobility exercises (e.g. standing trunk movements)<br>● Strengthening exercises (e.g. isometric wall squat, prone press-ups; plank; abdominal strengthening exercises)<br>● Active and passive stretching exercises (standing anterior thigh stretch)
Late Phase<br><br>Goals:<br>● Functional restoration of range of motion<br>● Return to daily activities<br>● Increase time of exposure to daily activities that elicit symptoms<br>● Exposure to more demanding daily activities<br>● Continue to improve physical fitness<br>● Foster independent symptom management	Add or increase external load of prescribed exercises (bands and/or free weights)<br><br>Increase complexity and add single leg stance exercises:<br><br>● Articular mobility exercises (e.g. single leg knee to chest)<br>● Strengthening exercises (e.g. plank with trunk movement or with hand movement; lunges)<br>● Active and controlled stretching exercises

Notes:

In addition to anamnesis, physical assessment comprised the evaluation of movement patterns, addressing compensations/difficulties during movements executions, and active and passive range of motion in uni-joint and multi-joint movements (measured by the motion trackers in the medical device).

The exercise prescription was tailored according to the initial assessment and individual patient progress during the study.

The following parameters of the exercise prescription were adjusted by the physical therapist according to the patient's evolution: range of motion, number of exercises, number of sets and repetitions and the type of exercise. Alongside assessment and communication between patients and PT, the performance (namely the range of motion, execution, movement compensations and skipped exercises) and the level of pain and fatigue during exercises reported by the patient were taken in consideration for the intervention decision making.

Lower limb and neural glide exercises were prioritized in the presence of radicular pain.

All necessary adjustments to the protocols were registered. Deviations from protocol required the provision of a clinical justification by the DPT, which was audited by the study PI.

Supplementary Figure 2. Biofeedback system provided to each participant, composed of 2 motion trackers and a tablet with a dedicated App displaying the prescribed exercise sessions.

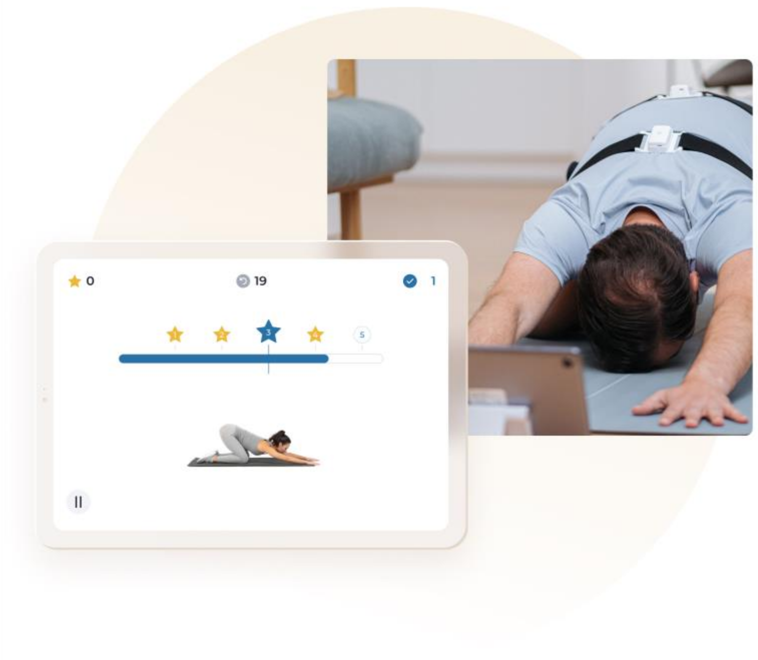

Supplementary Table 6. Description of exercise prescription in the conventional group.

Stages and Main Goals	Intervention Description
Initial Phase<br>Goals:<br>● Decrease symptoms intensity/severity and inflammatory signs<br>● Gradually exposure to avoided movements<br>● Improve range of motion (passive and active)<br>● Improve muscular strength and neuromuscular control<br>● Functional activity modification and exposure	Aim for 2-4 sets of exercises, including:<br>● Articular mobility exercises (e.g. lumbar rotation in hook-lying, prone extension)<br>● Active and passive stretches (e.g. gluteus/piriformis stretch, quadriceps stretch)<br>● Strengthening exercises (e.g. wall slides, bridges, quadruped opposite arm and leg extension, plank, abdominal strengthening)
Late Phase<br>Goals:<br>● Return to daily activities<br>● Functional restoration of range of motion<br>● Return to activities of daily living<br>● Improve overall condition	Increase exercise dosage (through repetitions and sets) and complexity, and add external load (bands and/or free weights):<br>● Articular mobility exercises (e.g. single leg knee to chest)<br>● Strengthening exercises (e.g. single leg bridges, prone opposite arm and leg extension on a balance ball, side plank, unsupported dead bugs)<br>● Active and passive stretches<br>Include additional sports/activity specific exercises.
Notes:<br>The exercise prescription was individualized according to the initial evaluation (including patient's symptoms and baseline physical capabilities) and the individual progress during the study. Gradual progressions on exercise dosage (sets, repetitions and intensity) were performed according to the patient's tolerance. Patients were instructed to continue to perform the exercises at home.	
